# Cognitive Functions in Elite and Sub-Elite Youth Soccer Players Aged 13 to 17 Years

**DOI:** 10.1371/journal.pone.0144580

**Published:** 2015-12-11

**Authors:** Barbara C. H. Huijgen, Sander Leemhuis, Niels M. Kok, Lot Verburgh, Jaap Oosterlaan, Marije T. Elferink-Gemser, Chris Visscher

**Affiliations:** 1 Center of Human Movement Sciences, University Medical Center Groningen, University of Groningen, Groningen, The Netherlands; 2 AZ Alkmaar, Alkmaar, The Netherlands; 3 Section Clinical Neuropsychology, VU Amsterdam, Amsterdam, The Netherlands; 4 Institute for Studies in Sports and Exercise, HAN, University of Applied Sciences Nijmegen, Nijmegen, The Netherlands; University of Rome "Foro Italico", ITALY

## Abstract

Soccer players are required to anticipate and react continuously in a changing, relatively unpredictable situation in the field. Cognitive functions might be important to be successful in soccer. The current study investigated the relationship between cognitive functions and performance level in elite and sub-elite youth soccer players aged 13–17 years. A total of 47 elite youth soccer players (mean age 15.5 years, SD = 0.9) and 41 sub-elite youth soccer players (mean age 15.2 years, SD = 1.2) performed tasks for “higher-level” cognitive functions measuring working memory (i.e., Visual Memory Span), inhibitory control (i.e., Stop-Signal Task), cognitive flexibility (i.e., Trail Making Test), and metacognition (i.e., Delis-Kaplan Executive Function System Design Fluency Test). “Lower-level” cognitive processes, i.e., reaction time and visuo-perceptual abilities, were also measured with the previous tasks. ANOVA’s showed that elite players outscored sub-elite players at the “higher-level” cognitive tasks only, especially on metacognition (p < .05). Using stepwise discriminant analysis, 62.5% of subjects was correctly assigned to one of the groups based on their metacognition, inhibitory control and cognitive flexibility performance. Controlling for training hours and academic level, MANCOVA’s showed differences in favor of the elite youth soccer players on inhibitory control (*p* = .001), and cognitive flexibility (*p* = .042), but not on metacognition (*p* = .27). No differences were found concerning working memory nor the “lower-level” cognitive processes (p > .05). In conclusion, elite youth soccer players have better inhibitory control, cognitive flexibility, and especially metacognition than their sub-elite counterparts. However, when training hours are taken into account, differences between elite and sub-elite youth soccer players remain apparent on inhibitory control and cognitive flexibility in contrast to metacognition. This highlights the need for longitudinal studies to further investigate the importance of “higher-level” cognitive functions for talent identification, talent development and performance in soccer.

## Introduction

In contemporary Western society, sports are part of our everyday lives with elite soccer having become a multi-million euro business. Early identification of talented soccer players is considered of high importance by many professional soccer clubs since they believe this enlarges the chances of an elite career. It is thought to enable selecting those players who have the potential to reach the elite level and as such, enables large focused investment of talent development programs. In this attempt to identify and develop young talented players, sports scientists work together with trainers, coaches and scouts to underline key elements of the talent identification and development process [1]. A well-known strategy is to compare successful with less successful soccer players on determining factors that seem important to achieve success [2–4]. The other side of the coin is that practicing soccer at sufficiently high level may represent an opportunity for adolescents to develop cognitive functions that are relevant for academic achievement and success later in life. E.g., Wang and colleagues [5] suggest that those who have difficulties in inhibitory control may benefit from sports with both physical and cognitive demands.

Previous research on talent identification in team sports concluded that the success in these sports depends on the level of multiple performance characteristics which can be either sport-specific or also transferable to other life settings, i.e., anthropometrical, physiological, technical, tactical and psychological performance characteristics [6–9]. During childhood and adolescence, talented players need to develop themselves in virtually every aspect of these multidimensional performance characteristics in order to reach the top [10]. Recent evidence suggests that success in soccer depends on how information is processed given the complex and quickly changing contexts [11] highlighting the importance of cognitive skills to play the game of soccer.

Open skill sports, such as soccer, are defined as those in which players need to react in a dynamically changing, unpredictable and externally-paced environment [5]. Therefore, soccer players may develop more flexibility in visual attention, decision making and action execution [12, 13]. Players are constantly searching for the best options. The choices players make, are based on the information that they recognize within the context of the match (i.e., ‘working memory’). A player must be able to quickly anticipate and react to the fast changing situations that occur during a soccer match. For example, the positioning of teammates and opponents are constantly changing during the match. When having the ball, the player could give a pass (long, short, forward, sideways, etc.), start a dribble or wait to make a move (i.e., cognitive flexibility’). Furthermore, a player should be able to cancel an intended pass to a teammate in case that teammate suddenly becomes defended. Therefore, players need the ability to quickly suppress their motor responses and make a new decision (‘inhibitory control’). In short, a player should be able to adapt his planned actions in such a way, that he makes a good, quick and effective decision based on the changes on the field.

A distinction can be made between “lower-lever” and the “higher-level” cognitive functions [14]. “Lower-level” cognitive processes are necessary for the basic information processing, such as reaction time, psychomotor performance and visuo-perceptual abilities [15]. Results of previous studies are incongruent when comparing performance levels on these “lower-level” cognitive processes. Most studies found no differences related to level of performance in sport [12, 16–19]. However, some studies found that elite athletes had significantly faster reaction times and psychomotor responses than novices and sub-elite players [20, 21].

The “higher-level” cognitive functions are often referred to as executive functions (EF) and are involved in the control and regulation of “lower-level”, non-EF cognitive processes, such as reaction time, EF enable goal-directed, future-oriented behavior [14]. Inhibitory control, cognitive flexibility, and working memory (i.e., holding information in mind and manipulating this information in memory), are known as the three ‘core’ EF [22–24]. The ability to adapt quickly to new demands and rules, based on the fast changing situations in the field indicates that cognitive skills like inhibitory control and cognitive flexibility could be of great importance concerning soccer-related skills. In addition, working memory may be important for choosing positions and mentalizing possible options in the game [25]. A combination of various EF is defined as a players’ metacognition. Metacognition reflects “the use of strategies that are thoughtfully brought to mind as one prepares to solve a problem and then a monitoring of progress towards a specific goal” [26]. The importance of EF and metacognition in adult soccer players and young soccer players is already shown in recent studies [11, 25]. High Division soccer players outperformed the Low Division players on multiple EF tasks, measuring working memory, inhibitory control, and cognitive flexibility [11]. Highly talented soccer players on average aged 11 outscored their amateur counterparts on motor inhibition and alerting [25].

So far, no studies in soccer investigated the combination of the “lower-level” and “higher-level” cognitive processes, and no studies addressed the distinction between core-EF and metacognition within the “higher-level”. In view of talent identification and development in soccer it seems important to acquire knowledge on various cognitive processes in relation to performance. Lower level cognitive processes are considered a prerequisite to play soccer, and as such, are not expected to differ between players from a talent development program from a professional club and amateur youth players. However, based on previous research as described above, higher level cognitive processes are hypothesized to be better in players from a talent development program compared to amateur players. Possible differences may be related to a difference in training hours between players of distinctive levels of performance. In addition, less research has been conducted with talented soccer players aged around 15, a highly relevant age in soccer since at this age player may be offered professional contracts. Fifteen is also the age at which maturation of EF accelerates [27]. Elementary forms of EF are present early during the preschool period (3–5 years) and develop from early childhood through adolescence into young adulthood [28–30]. EF might be related to intelligence, it is shown that, academic level can be a good predictor of intelligence [31].

The goal of the present study was to explore cognitive functions in elite and sub-elite youth soccer players aged 13–17 years The players were compared on tasks measuring “lower-level” cognitive processes (i.e., reaction time and visuo-perceptual abilities) as well as “higher-level” cognitive functions (executive functions; EF). These include the core EFs (i.e., working memory, inhibitory control, and cognitive flexibility), and metacognition The study controlled for the effects of age, training hours, and academic level. Our hypothesis is that cognitive differences between elite and sub-elite youth soccer players are observed in the “higher-level” cognitive functions (i.e., EF), not on the “lower-level” processes.

## Method

### Participants

A total of 88 male soccer players from the Netherlands, aged 13–17 years, participated in this study. The 47 elite youth soccer players (mean age 15.48 years, SD = 0.90) played in the highest competitive level for their age group and belonged to the top 0,5% of all players (National Soccer Association, KNVB). They were part of a talent development program of a professional soccer club. The group of sub-elite youth soccer players consisted of 41 soccer players (mean age 15.15 years, SD = 1.18) who played in the top 12,5% of all players in their age category (National Soccer Association, KNVB). Participants reported on the number of years of soccer training they have had and the current weekly number of training hours. Furthermore, they reported on the average weekly number of soccer matches (Table 1).

**Table 1:** Characteristics of the elite and sub-elite youth soccer players (mean ± SD).

	Elite youth soccer players (n = 47)	Sub-elite youth soccer players (n = 41)	p-value
Age (years)	15.5 (0.9)	15.2 (1.2)	0.142
Training (h / week)	10.7 (1.0)	2.9 (1.1)	<0.001
Matches (h / week)	1.0 (0.0)	1.0 (0.2)	0.287
Soccer experience (years)	9.8 (0.8)	9.3 (1.5)	0.035
**Academic level**			
Pre-university (n [%])	36 (76.6)	17 (41.5)	0.001
Pre-vocational (n [%])	11 (23.4)	24 (58.5)	

*Note*: The national average of students at pre-university academic level is 57.9% and at pre-vocational academic level is 42.1% in the Netherlands [32].

### Materials

To measure lower-level and higher-level cognitive functions, various measures were obtained. Lower-level cognitive functions were measured with the Trail Making Test (visual perceptual ability; TMT-A) and with the Stop-Signal task (reaction time; MRT). Core executive functions were measured with the Backward Visual Memory Span (working memory; Backward VMS), the Stop-Signal Task (inhibitory control; SSRT) and the Trail Making Test (cognitive flexibility; B-A difference). Metacognition was measured with the D-KEFS Design Fluency Task (DFT).

#### Trail Making Test (TMT)

The Trail Making Test (TMT) consisted of two parts (A and B) [15]. The TMT-A is commonly used as a measure for visuo-perceptual abilities, the “lower-level” cognitive task. The TMT-B is used as a measure for cognitive flexibility [33]. In both parts the test consisted of 25 circles distributed over a sheet of paper. In TMT-A, the circles were numbered 1–25, and the participant had to draw lines to connect the numbers in ascending order. In TMT-B, the circles included both numbers (1–13) and letters (A–L); the participant had to draw lines to connect the circles in an ascending pattern, alternating between the numbers and letters (i.e., 1-A-2-B-3-C, etc.). By subtracting the total time of TMT-A from the total time of TMT-B and thereby removing the motor component in the score of the TMT-B, a more objective and valid measure for cognitive flexibility was obtained [15, 34]. A smaller B-A difference, indicates better cognitive flexibility. TMT’s reliability is moderate in youth sport participants (r = .71) [35].

#### Stop-Signal Task (SST)

The Stop-Signal task (SST) was used to measure motor inhibition of an ongoing response (i.e., inhibitory control) [36]. The task involved go-trials and stop-trials. The first part only consisted of go trials, in which the participants had to respond as quickly and accurately as possible to a go-stimulus (cartoon airplane presented for 1000ms). This part of the task, defined as the mean reaction time (MRT) is considered the “lower-level” cognitive task. In the stop-trials the players had to inhibit the response if a stop-stimulus (cross presented for 50ms) was presented. The initial delay between the cartoon airplane and the cross was 175 ms. The delay was increased by 50 ms if the participant inhibited the response, and decreased by 50 ms if the participant failed to inhibit the response. The inter-trial interval was 1500 ms. Two practice blocks of 32 trials, of which the first included only go-trials, and the second included go-trials and stop-trials, preceded three experimental blocks of 64 trials of which 25% were stop-trials. The stop signal reaction time (SSRT) is an estimate of the time a participant needed to stop his or her response (MRT) minus the mean delay, with shorter SSRTs indicating better inhibitory control [36, 37]. High reliability of SSRT and MRT is reported for the age of 13–17 years (split-half reliability = .91 and .93, respectively) [38].

#### Backward Visual Memory Span (VMS)

The Backward Visual Memory Span (VMS) was used to measure working memory. This test requires to hold information in mind, and manipulating that information in mind [39]. The examiner tapped a certain number of squares that were printed in a particular order on a sheet of paper. The participant was required to repeat the sequence in the reverse order (i.e,. backwards). The eight squares were labeled with numbers that were only visible to the examiner. The task began with two sequences of 2 units and, if the participant succeeded, sequences of increasing length were presented (i.e., two sequences of 3 units, two sequences of 4 units, etc.). The task ended when a participant failed on two sequences of the same length. The dependent variable is the length of the longest sequence (step 1–7) tapped correctly, with longer sequences indicating better working memory. A high reliability of the backward VMS is reported for the subjects aged 16–17 years (r = .82) [40].

#### D-KEFS Design Fluency Test (DFT)

The D-KEFS Design Fluency Test (DFT) is a standardized test which measures working memory, inhibitory control, and cognitive flexibility, as well as creativity and planning [11, 41, 42]. It comprises three conditions: filled dots, empty dots, and switching. In each condition, participants were instructed to make a different design in each square by connecting dots for 60 s using only four straight lines. For each of the three conditions, the player had 60 seconds to draw as many different designs by connecting using only four straight lines. The total number of correct and unique designs were analyzed and scored by the test leader on the test form. No credit was given if the design contains greater or fewer than four lines or was a repetition of a previous design [41, 42]. In the first condition, the participants were presented squares containing five filled (i.e., black) dots, and they were asked to draw their designs by connecting the dots. In the second condition, the squares contained five empty dots and five filled dots. Participants were instructed to connect only the empty dots. In the third and final condition, the squares also contained five empty dots and five filled dots; however, in this condition, the participants were instructed to alternate between connecting empty dots and filled dots. Credit was not given for designs in which participants did not switch correctly. Test-retest reliability has been found moderate [41].

### Procedure

The tasks were administered in a 40-minutes session. The tests were administered by the first author of the paper, a well-trained, certified test leader of a high-academic level and an expert in testing executive functioning in sport contexts. The participants performed four cognitive tasks in a fixed order: Visual Memory Span (VMS), Stop-Signal Task (SST), Trail Making Test (TMT) and the D-KEFS Design Fluency Test (DFT). Responses were collected and analyzed anonymously by the first author of the paper.

The tasks were conducted halfway through the competitive season (January to March). Responses for each test were collected according to the manual [11, 34–36]. This is explained in short for each test below: Visual Memory Span (VMS):, the score is given and scored on the test form by the test leader. Stop-Signal task (SST): This test is executed electronically, the computer collected all data. Trail Making Test (TMT): This test was executed on a test form (paper). The test leader timed the test and this score was written down on the test form. D-KEFS Design Fluency Test (DFT): This test was executed on a test form (paper).

The current study did not fall under the scope of the Medical Research Involving Human Subjects Act (WMO) and was therefore not reviewed by an accredited MREC or the CCMO. However, to meet the ethical standards the following procedure was undergone. First, the 2 soccer clubs and the involved coaches were informed about the procedures, the clubs agreed to participate in the study. Second, the parents / guardians and players were given information about the research by an information meeting as well as by an information letter. All involved parties were informed before participation and signing the informed consent with the following information:

- A statement that the study involves research
- Explanation of the purposes of the research
- Names of the principal researchers
- Expected duration of the subject's participation (40 min)
- Description of the procedures to be followed
- Description of any reasonably foreseeable risks or discomforts to the subject (the study is considered as minimal risk)
- Explanation of whom to contact for answers to pertinent questions about the research and research subjects' rights
- Statement that participation is voluntary, their right to decline to participate and to withdraw from the research once participation has begun and the subject may discontinue participation at any time without penalty or loss of benefits to which the subject is otherwise entitled.

Only players and parents who signed the informed consent were included in the study.

### Statistical analysis

Data were analyzed using IBM SPSS Statistics 20.0.0 (IBM Corp. Somers, NY). To check normality, z-scores of skewness and kurtosis of each variable were calculated. Results showed z-scores between -2.58 and 2.58. Therefore, the data were considered to be indicative of an approximately normal distribution [43]. Means scores and standard deviations of the measures cognitive functions were calculated separately for the elite and sub-elite youth soccer players. Separate ANOVA’s are performed to analyze possible differences between elite and sub-elite youth soccer players on the descriptive characteristics as well on the outcome variables. To interpret the scores on the outcome variables, the Cohen’s *d* effect sizes were calculated(*d*) between the elite and sub-elite youth soccer players. An effect size around or below 0.20 is considered small, around 0.50 medium, and around 0.80 large [44]. To identify discriminating variables between elite and sub-elite youth soccer players, a stepwise discriminant analysis was performed. In this analysis, level of performance (elite or sub-elite) was the dependent variable and the outcome variables of the cognitive tasks were the independent variables. The academic system in the Netherlands is divided into two systems: the pre-university system, in which students are prepared for a university career, or the pre-vocational system, in which students are prepared for later vocational education. Since EF may be related to academic level, and weekly training hours these variables were used as covariates. Two multivariate analysis of covariance (MANCOVA’s) were performed to examine differences in the “lower-level” cognitive tasks and the “higher-level” cognitive tasks between both groups while taking into account the covariates academic level and weekly training hours. For the first MANCOVA the two variables form the “lower-level” cognitive tasks (TMT-A and MRT) served as the dependent variables, whereas level of performance (elite or sub-elite) was included as the between-subjects independent variable. The second MANCOVA included the four EF variables (Backward VMS, SSRT, B-A difference, and DFT).

Differences between elite youth soccer players and sub-elite youth soccer players on the scores of the “lower-level” cognitive tasks and the “higher-level” EF tasks, were determined by comparing means using a univariate analysis of variance (ANOVA) or covariance (ANCOVA), depending on the results of the multivariate analysis. Statistical significance was accepted at *p* < .05.

## Results

The χ^2^-test revealed a significant difference in academic level between the elite and sub-elite groups [χ^2^(1, 84) = 11.28, *p* = .001]. A significantly higher percentage of elite youth soccer players were enrolled in the pre-university system compared to sub-elite youth soccer players (Table 1). In addition, a significant difference was found between elite and sub-elite youth soccer players in terms of weekly training hours (*p* < .01).

The means and standard deviations for both groups on the “lower-level” cognitive tasks and “higher-level” EF tasks are shown in Table 2. Significant differences were found between elite and sub-elite players at the “higher-level” EF tasks of the SSRT, the TMT B-A difference and on the DFT.

**Table 2:** Scores (mean ± SD) of the “lower-level” cognitive tasks and EF tasks scores of elite (n = 47) and sub-elite (n = 41) youth soccer players.

	Eliten = 47	Sub-eliten = 41	Group effects (p-value)	Effect size of group effect (d)
**“Lower-level” tasks**				
TMT-A (s)^a^	26.9 ± 9.9	30.1 ± 11.8	.171	0.29
MRT (ms)^a^	424.1± 89.6	430.3 ± 73.2	.725	0.08
**“Higher-level” EF tasks**				
Backward VMS^b^	5.3 ± 1.2	4.9 ± 1.1	.144	0.35
SSRT (ms)^a^	197.5 ± 37.1	216.3 ± 33.6	.015	0.53
B-A difference (s)^a^	32.1 ± 17.7	43.8 ± 25.8	.014	0.53
DFT^b^	37.1 ± 7.8	33.0 ± 6.0	.007	0.60

^a^ Lower scores indicate better performance.

^b^ Higher scores indicate better performance.

The stepwise discriminant analysis showed that the combination of three higher-level cognitive functions, i.e., DFT, SSRT and TMT B-A difference scores best discriminated between playing level (Table 3). No lower-level cognitive functions entered the model. The model demonstrated that the DFT score best discriminated between the elite and sub-elite youth soccer players with a standardized discriminant function coefficient of .622, followed by a negative coefficient of -.537 for SSRT and a negative coefficient of -.510 for TMT B-A. Negative coefficients indicate lower scores representing better performance (i.e., less time needed for the test). The average squared canonical correlation was .422, showing that these three variables, accounted for 42.2% of the overall variance in the data set. The three variables correctly classified 62.5% of the players as either being part of the elite or sub-elite group.

**Table 3:** Stepwise discriminant analysis of the outcome variables of the cognitive tasks.

			Exact F			
Step	Entered	Lambda	Statistic	df1	df2	p-value
1	DFT	.919	7.56	1	86	.007
2	SSRT	.862	6.83	2	85	.002
3	TMT B-A	.822	6.06	3	84	.001

*Note*: at each step, the variables that minimizes the overall Wilks’ lambda is entered. Minimum partial *F* to enter is 3.84, maximum *F* to remove is 2.71.

The MANCOVA for the “lower-level” cognitive tasks revealed no significant main effect for group [Wilks’ λ = .96, *F*(2,83) = 1.80, *p* = .172], indicating no differences between the elite and sub-elite youth soccer players. Academic level was found significant for the “lower-level” cognitive tasks [Wilks’ λ = .91, *F*(2,83) = 3.94, *p* = .023], indicating better performance for soccer players enrolled in the pre-university system compared to players in the pre-vocational system. Training hours per week were not found a significant covariate for the “lower-level” cognitive tasks [Wilks’ λ = .95, *F*(2,83) = 2.21, *p* = .116].

The MANCOVA for the EF tasks revealed a significant main effect for level of performance [Wilks’ λ = .82, *F*(4,81) = 4.55, *p* = .002], indicating better overall performance of the elite youth soccer players compared to sub-elite youth soccer players. No significant differences were found for academic level on the EF tasks [Wilks’ λ = .95, *F*(4,81) = 1.00, *p* = .410]. Training hours per week were found as a significant covariate [Wilks’ λ = .87, *F*(4,81) = 2.99, *p* = .023]. The analysis for the EF tasks was followed by a univariate ANCOVA on each of the four dependent variables with level of performance as independent variable and training hours as covariate.

Concerning the EF tasks, controlling for training hours, no significant difference between groups was found on the working memory task (i.e., backward VMS) [*F*(1,84) = 1.711, *p* = .194]. In contrast, elite youth soccer players showed faster SSRT’s indicating better inhibitory control than sub-elite youth soccer players [*F*(1,84) = 4.25, *p* = .042] regardless of the covariate weekly training hours. Similar results were found on the TMT B-A difference indicating better cognitive flexibility of the elite youth soccer players [*F*(1,84) = 11.70, *p* = .001]. The significant group differences translated into medium sized effect. Finally, with training hours as covariate, no significant difference was found anymore between elite youth soccer players and sub-elite youth soccer players concerning the DFT, which provides an aggregated measure of working memory, inhibitory control, and cognitive flexibility [*F*(1,84) = 1.22, *p* = .273], indicating metacognition.

## Discussion

To better understand the characteristics of those players who have been selected for a talent development program aiming to increase their chance to reach the elite level and as such, to enable large focused investment of talent development programs, the goal of the current study was to explore cognitive functions in elite and sub-elite youth soccer players. Elite and sub-elite players aged 13 to 17 were compared on “lower-level” cognitive tasks as well as on “higher-level” EF tasks which measured the core EFs working memory, inhibitory control, cognitive flexibility, as well as metacognition. Our study showed that elite youth soccer players outperformed sub-elite youth soccer players on metacognition, inhibitory control, and cognitive flexibility, but not on “lower-level” cognitive functions. Differences between elite and sub-elite youth soccer players on metacognition were no longer significant when taking into account the number of training hours, whereas differences on inhibitory control and cognitive flexibility remain apparent.

Although the results are based on a cross-sectional study, and await replication in a design that allows causal inferences to be made our findings reveal a possible explanation for differences in performance between youth soccer players in terms of their cognitive functions. In terms of talent identification in youth soccer, the lower-level cognitive functions do not seem useful. However, the core executive functions inhibitory control and cognitive flexibility may prove relevant for talent identification purposes, especially since these functions are not significantly to influenced by the number of training hours. Considering the ‘other side of the coin’, in terms of talent development, playing soccer at a high level of performance each day, i.e., in a talent selection team of a professional soccer club, seems to be related to the level of metacognitive skills. Although in the current study we only included the number of training hours in our analyses, it is hypothesized that it is not just the quantity but even more so the quality of training which is important in this respect. For this, additional research is recommended.

The current study extends the findings of previous studies on executive functioning which indicated that elite athletes outperform the sub-elite athletes [11, 25], to adolescent soccer players, indicating the importance of executive functioning in soccer at a young age. The stepwise discriminant analysis revealed that the higher-level cognitive functions metacognition (DFT), inhibitory control (SSRT) and cognitive flexibility (TMT B-A difference) accounted for 42.2% of the overall variance in the data set, with the remaining likely to be made up from other variables associated with soccer performance (e.g., technical and tactical skills). The three higher-level cognitive functions together accounted for 62,5% of the correct classification between elite and sub-elite players, this adds 12,5% to classification based on chance (50%). We assume that this 12,5% is relevant, as we compared within a group of experienced soccer players

No differences were found on the “lower-level” cognitive processes concerning basic information processing such as reaction time and visuo-perceptual abilities. These findings strengthen the idea that there is a specific relationship between “higher-level” cognitive functions and the level of performance in youth soccer. Previous research already showed that accuracy in combination with speed is more important than speed itself. In comparing successful youth players who were allowed to stay in a talent development program of a professional soccer club with the less-successful ones who were released from the program, it was found that players were equally fast on a soccer passing-test on the field, however, the successful players made less mistakes [45].

Results of the working memory task (backward VMS) indicated no group differences. An explanation may be that the VMS, similar to The Corsi Block test, is a simple memory span task using only the storage component of working memory and not the control function of working memory [46]. Previous research showed that differences between elite and sub-elite youth players were mainly found on procedural knowledge, on “knowing what to do and when to execute the appropriate action” and not so much on stored knowledge, i.e., declarative knowledge [47, 48]. In line with this, a recent study in elite youth soccer players showed no difference with amateur soccer players on visuo-spatial working memory either [25]. Also no differences in visuo-spatial working memory between adult basketball players and non-athletes were found [49].

In line with our hypotheses, elite youth soccer players outperformed sub-elite youth soccer players on inhibitory control, but do not have faster reaction times. Our findings show that the differences between elite and sub-elite youth soccer players concerning cognitive skills, are not to be explained in terms of “lower-level” processes, but on the “higher-level”, the more complex executive functions. The current findings provide a first insight in the age group from 13–17 years in inhibitory control of motor responses between elite and sub-elite adolescent soccer players. The ability to quickly inhibit motor responses during important soccer-related skills seems a necessity to be a successful youth soccer player. During a soccer match, players are required to quickly react to teammates and opponents, and in addition withhold executing an intentional pass, dribble or shot if this is no longer possible due to the changing situations in the field. These results are supported by previous studies that found similar results between professional volleyball players and non-athletes and high-skilled and lower-skilled baseball players [16, 17]. Furthermore, comparable results were found on motor inhibition skills in youth elite soccer players (mean age 11.9 years) [25].

On the Trail Making Test, elite youth soccer players outperformed sub-elite soccer players on B-A difference, a measure for cognitive flexibility, with no differences between both groups on TMT-A, a measure for the “lower-level” cognitive process visuo-perceptual abilities. This study is the first to demonstrate better cognitive flexibility skills in elite soccer players at young age. In order to adapt quickly to new demands, rules, or priorities based on the fast changing situations in the field, high visuo-perceptual ability is not sufficient. Players need to excel in cognitive flexibility which underlines the importance of “higher-level” cognitive processes in soccer. Concerning metacognition, an aggregated measure of working memory, inhibitory control and cognitive flexibility was used, on which elite youth soccer players outperformed sub-elite soccer players. This finding was also reported in High Division soccer players comparing Low Division soccer players on the DFT [25]. In the metacognitive task a creativity aspect in solving the problem was included [11]. In general, creativity is defined as “the ability to produce work that is both novel (i.e., original, unexpected) and appropriate (i.e., useful)” [50, 51]. In soccer, creativity refers to those varying, rare, and flexible decisions that are important for performance [52]. The significant effect of weekly number of training hours as a covariate in the comparison between elite and sub-elite youth soccer players on metacognition is in line with findings from studies on the relation between creativity and expertise [53, 54] and suggests that the explanation of the results can go both ways. Elite players may perform better because of their superior metacognition, however, an alternative explanation may also hold, i.e., because of their training hours at the elite level, elite players develop their metacognition to a greater extent. These findings are highly relevant for those who are interested in the potential of soccer to promote EF development in adolescence and extent to the literature on trainability and transferability of ‘higher-level’ cognitive functions to for example education. (e.g. [55–57]).

A limitation of the current study is the cross-sectional design. EF matures up till young adulthood (i.e., 21 years). The development of EF over time is different for each individual [28]. Therefore, to further unravel the importance of EF in soccer, a longitudinal design is recommended in which youth athletes are monitored throughout their sports career. Differences between elite and sub-elite players in cognitive functions may be due to the quality and duration of the practice/match play. However, the literature concerning the effects of training on EF in other domains is inconsistent [56–59], this may also be the case in soccer. EF is at least partly genetically determined [60] and elite youth soccer players already outscore their sub-elite counterparts as young as 8 years, when they first enter talent development programs [25]. Therefore, differences in EF skills between elite and sub-elite players may have already emerged before the extra amount of practice or match play in talent development programs.

In sum, the current study investigated cognitive functioning in elite and sub-elite youth soccer players. We showed that with similar reaction times and visuo-perceptual abilities, elite youth soccer players have better inhibitory control, cognitive flexibility and, especially, metacognition than their sub-elite counterparts. When weekly training hours are taken into account, differences between elite and sub-elite youth soccer players remain apparent on inhibitory control and cognitive flexibility in contrast to metacognition. This highlights the need for longitudinal studies to further investigate the importance of “higher-level” cognitive functions for talent identification, talent development and performance in soccer.
